# Ecological roles of secondary metabolites of *Saposhnikovia divaricata* in adaptation to drought stress

**DOI:** 10.7717/peerj.14336

**Published:** 2022-11-04

**Authors:** Sisi Cao, Lei Shi, Ying Shen, Luwen He, Xiangcai Meng

**Affiliations:** 1 Department of Pharmacognosy, Heilongjiang University of Chinese Medicine, Harbin, Heilongjiang, China; 2 Medical College, Harbin Vocational & Technical College, Harbin, Heilongjiang, China

**Keywords:** *Saposhnikovia divaricata* (Turcz.) Schischk, Metabolomics, Reactive oxygen species, Polyethylene glycol-6000, Secondary metabolites, Metabolic pathways, Antioxidant enzymes

## Abstract

*Saposhnikovia divaricata* is a traditional Chinese herb that mainly grows in arid grasslands and strongly adapts to various stresses. Drought is not only a major abiotic stress factor but also a typical feature conducive to producing high-quality medicinal material. The present study investigated by treating *S. divaricata* plants with polyethylene glycol (PEG-6000). Ultra-high performance liquid chromatography-quadrupole time-of-flight mass spectrometry (UPLC-Q-TOF-MS) identified 146 compounds from the roots of *S. divaricata*, among which seven primary metabolites and 28 secondary metabolites showed significant changes after drought treatment. UV-Vis spectrophotometer detected the activity of antioxidant enzymes and the content of superoxide anion (O_2_^−.^) and *malondialdehyde* (MDA). The differential primary metabolites revealed that drought promotes glycolysis, reducing primary metabolism and enhancing secondary metabolism. Meanwhile, the differential secondary metabolites showed an increase in the content of compounds upstream of the secondary metabolic pathway, and other glycosides and increased that of the corresponding aglycones. The activities of antioxidant enzymes and the content of O_2_^−.^ and MDA shown different changes duing the drought treatment. These observations indicate that drought promotes the biosynthesis and transformation of the secondary metabolites and activity of antioxidant enzymes, improving plant adaptability. The present study also analyzed a few primary and secondary metabolites of *S. divaricata* under different degrees and durations of drought and speculated on the metabolic pathways in an arid environment. The findings indicate the biological nature, diversity, and complexity of secondary metabolites and the mechanisms of plant adaptation to ecological stress.

## Introduction

Abiotic stresses, namely drought, heat, salinity, coldness, and pathogen infection represent the major limitations on biological growth. Therefore, plants and animals must evolve numerous mechanisms to accommodate with changes of stress. During the prolonged evolutionary process, plants and animals adopted different adaptation strategies and were divided into two mutually distinct taxa (Ronald & Beutler, 2010). Animals can move and migrate to different locations and stay protected from a harsh environment, but plants cannot move and hence face severe abiotic stresses, such as drought, heavy metals, and high salinity (Ronald & Beutler, 2010; Rehman et al., 2021), which affect their growth, metabolism, and yield (Zhu, 2016).

Signal molecules, transcription factors, genes, and defense components are activated under stress, triggering complex plant responses (Mittler, 2005). Meanwhile, plants absorb more light energy than that required for carbon dioxide (CO_2_) fixation, resulting in the reduction of excess O_2_ to superoxide anion (O_2_^−.^) (Strizh, 2008). Besides, a part of O_2_^−.^ is transformed into hydroxyl radical (·OH) and hydrogen peroxide (H_2_O_2_) (Zorov, Juhaszova & Sollott, 2014; Bodega et al., 2019). These substances are called reactive oxygen species (ROS) due to their strong oxidizing properties (Li et al., 2021; Li et al., 2015). Plants are equipped with a unique antioxidant system, which can maintain ROS within an appropriate range by regulating the oxidation–reduction balance (Kibria et al., 2017; Duan et al., 2022). This system is composed of enzymatic and nonenzymatic components, which prevent or delay cell damage by eliminating or inhibiting the oxidation of ROS (Dumont & Rivoal, 2019).

The enzymatic antioxidant defense system comprises superoxide dismutase (SOD), catalase (CAT), and peroxidase (POD) (Sachdev et al., 2021), which eliminate these ROS in animals, plants, and fungi (Staerck et al., 2017). The first formed O_2_^−.^ gets converted into H_2_O_2_ spontaneously or *via* SOD and then into O_2_ and H_2_O_2_ by CAT or POD (Wang et al., 2018). However, antioxidant enzymes are also proteins, which can be altered *via* excess ROS (Datir, Singh & Joshi, 2020; Attia et al., 2021). Therefore, plants use their nonenzymatic systems, which cooperate with the antioxidant enzymes to resist adversity (Pisoschi & Pop, 2015).

Plant metabolites are an adaptation to cope with complex, stressful environments (Erb & Kliebenstein, 2020). Many secondary metabolites (SMs) have been biosynthesized from primary metabolites (PMs) and accumulated in plant cells. Generally, a plant contains multiple SMs of the same category, which are affected by both the growth environment and the metabolic pathways (Singer, Crowley & Thompson, 2003; Li et al., 2020). Humans have also artificially synthesized SMs as a source of bioactive compounds directly used as herbal medicine or preparing modern drugs for treating diseases. To date, about 25% of the drugs have been obtained from medicinal plants (De Luca et al., 2012; Wurtzel & Kutchan, 2016). More than 2,140,000 of these plants display great diversity in structure, function, and biosynthesis (Thirumurugan et al., 2018). Many studies have showed that the elicitation based on the biotic or abiotic stressor stimulation *in vitro* was the most effective technology strategy to improve the production of SMs from medicinal plants (Isah, 2015; Isah et al., 2017; Pant, Pandey & Dall’Acqua, 2021). However, improving SM production through biotic or abiotic stress stimulation is needed to support the application of this technology (Ghosh et al., 2018; Al Hassan et al., 2017). Liquid chromatography–mass spectrometry (LC–MS) is the most employed technique in plant metabolomics under stress conditions (Tian et al., 2020; Schrimpe-Rutledge et al., 2016; Ghatak, Chaturvedi & Weckwerth, 2017).

Drought is the most common abiotic stress and the main factor leading to the mass production of ROS in plants (Gepstein, Grover & Blumwald, 2008; Ozturk et al., 2021), which significantly changes the physiological and biochemical indicators such as antioxidant enzymes and metabolites (Jogawat et al., 2021; Ozturk et al., 2021; Zahedi, Karimi & Venditti, 2021) and affects the accumulation of metabolites in medicinal plants. Studies on the germination and physiological characteristics of maize (*Zea mays*) under drought and salinity stresses, found that O^−.^_2_ and MDA levels and the activities of antioxidant enzymes such as SOD, POD, and CAT increased under these stresses (Yao et al., 2016). The water content decreased with increasing PEG concentrations in maize cultivars (Mohammadkhani & Heidari, 2007). A study on the effects of drought stress on the physiological, biochemical, and chemical components of *Cinnamomum cassia* seedlings revealed certain drought tolerance potential of the plants under short-term or mild drought stress; however, when the drought exceeded a certain degree, the physiological metabolism of seedlings was unbalanced (Zhong et al., 2021).

*Saposhnikovia divaricata* is one of the most commonly used herbal medicines in Asian countries, with excellent analgesic, antipyretic, and anti-inflammatory effects (State Pharmacopoeia Committee, 2020). Wild *S. divaricata* is mainly distributed in northeast China, Inner Mongolia, Hebei, Shandong, Henan, Shaanxi, and Gansu (Feng et al., 2021). The wild *S. divaricata* plants produced in Heilongjiang Daqing, Qiqihar, and Inner Mongolia have the best quality due to the high-efficacy content and large yield of medicinal materials (Wang et al., 2020a; Yang et al., 2020). Most high-quality producing areas of wild *S. divaricata* are grassland areas with adverse environmental conditions, such as slight annual rainfall, arid climate, and large temperature difference between four seasons (Men et al., 2018; Han et al., 2017). *S. divaricata* is characterized by “taproot” and “chrysanthemum heart” (State Pharmacopoeia Committee, 2020), which is the typical plant feature in a drought condition (Jiang et al., 2018; Nishihara et al., 2018). Studies showed that the main active components of *S. divaricata* were chromones and coumarins (Okuyama et al., 2001; Batsukh et al., 2021; Zhao et al., 2010; Yang et al., 2017). Although cimifugin 7-glucoside and *5-O-*methylvisamicin are the major compounds (State Pharmacopoeia Committee, 2020), cimifugin is the primary efficacy component (Jiang et al., 2018). Glycosides such as cimifugin 7-glucoside get converted into cimifugin and absorbed into the blood and play antipyretic, analgesic, and anti-inflammatory roles in the human body (Yang et al., 2017; Lee et al., 2020). The content of cimifugin is two to three times that of cultivated products, and hence the quality of wild *S. divaricata* is better (Wang et al., 2017; Zhao et al., 2014; Li, 2011). The moderate use of drought stress technology in the cultivated *S. divaricata* can effectively improve the accumulation of metabolites and the quality of medicinal materials.

The current research on *S. divaricata* focuses on the pharmacological effects (Zhao et al., 2010; Chun et al., 2016; Kong et al., 2013; Wang et al., 2017), separation and purification method of main components (Nishihara et al., 2018; Zhang et al., 2008; Li et al., 2006), such as chromones and coumarins (Ma et al., 2018; Sun et al., 2022; Yang et al., 2015; Chen et al., 2018), genome (Liu et al., 2018), and antioxidant activity (Kamino et al., 2016). However, the effects of *S. divaricata* on antioxidation and metabolites under drought stress have not been explored yet. The quality of herbal medicine is closely related to the diversity and complexity of SMs, which determines the effect of moderate ecological stress in medicinal plants. The present study used different concentrations of polyethylene glycol (PEG-6000) to simulate drought stress in *S. divaricata* plants (Shen et al., 2020; Lau et al., 2021; Meher et al., 2018) and explored the changes in metabolites, ROS, MDA, and antioxidant enzymes. It provided a basis for analyzing the adaptability of antioxidant enzymes and metabolites of *S. divaricata* under plant stress, and clarifying the effects of *S. divaricata* under drought stress. The study mainly indicated the biological nature, diversity, and complexity of metabolites and antioxidant enzymes and the mechanisms of plant adaptation to stress.

## Materials and Methods

### Plant materials

Two-year-old *S. divaricata* (Umbelliferae) plants were grown in the Medical Botanical Garden of Heilongjiang University of Chinese Medicine (126°38′E, 4543′N) in June 2018 and identified by Prof. Xiang-Cai Meng, College of Pharmacy, Heilongjiang University of Chinese Medicine. All the *S. divaricata* plants were grown under standard field conditions, with an average day/night temperature of 16 °C/4 °C and 14/10-h photoperiod.

In this experiment, PEG-6000 was used to simulate drought stress (Fuchino et al., 2021; Hellal et al., 2017; Arun et al., 2020). The Hogland’s nutrient solution was prepared, and different concentrations of PEG-6000 were added to simulate the drought stress (Yao et al., 2016; Mohammadkhani & Heidari, 2007; Zhong et al., 2021; Meher et al., 2018; Arun et al., 2020; Chen et al., 2020). A total of 48 *S. divaricata* plants were collected, and their roots were washed with running water. The *S. divaricata* plants were placed in the Hoagland’s solution for 7 days for adaptive culture and then divided into three experimental groups: thick (12–15 cm), medium (8–12 cm), and thin (4–8 cm). The samples were divided into blank, mild (10% PEG-6000), moderate (15% PEG-6000), and severe (30% PEG-6000) drought treatment groups. The transplanted *S. divaricata* plants were maintained in a greenhouse under constant 16/8-h photoperiod, 14,000 lx/day of light intensity, 23 ± 1 °C/18 ± 1 °C day/night temperatures, and 45–55% humidity. The samples were collected 0, 2, 4, 6, and 8 days after the drought treatment. Before sampling, the top 1.5 cm was removed after the oxidation of the previous sampling. The root samples obtained from the thick, medium, and thin plants were pooled, and the xylem was scraped off for further treatment.

The *S. divaricata* samples adopted in this paper are collected from the Medicinal Botanical Garden of Heilongjiang University of Chinese Medicine. Field experiments were approved by Heilongjiang University of Chinese Medicine (No. 20210113). and causes no damage to the environment.

### Sample preparation using ultra-high-performance liquid chromatography-quadrupole time-of-flight mass spectrometry

The extraction method referred to the previous research of the group, and the target extracts were chromones, coumarins, flavones, and other compounds (Huo et al., 2021; Liu et al., 2018). Approximately 500 mg of sample powder was weighed into a 50-mL centrifuge tube and extracted using 25 mL of methanol (Fischer Scientific, Horsham, UK) at 80 °C twice, each for 1 h. The samples were centrifuged at 4 °C and 14,000*g* for 10 min, and the supernatant was collected into a derivatized glass bottle and freeze-dried. All the dried extracts were dissolved in methanol (1 mL) and filtered through a 0.22-μm Millipore filter, and the filtrate was collected for ultra-high-performance liquid chromatography–mass spectrometry (UPLC) analysis. Each sample was assayed three times.

### Sample preparation for superoxide anion (O_2_^−.^), MDA, and antioxidant enzyme analyses

The ice bath environment was prepared before sample processing, and all the vessels and test solutions in contact were refrigerated. The sample processing was carried out in an ice bath environment of 0 °C. Then, the 0.2000 g *S. divaricata* sample was accurately weighed into a mortar, mixed with 2 mL of normal saline (Harbin Medisan Pharmaceutical Co., Ltd, Harbin, China), and ground evenly. The sample suspension was transferred into a 5-mL centrifuge tube and centrifuged at 4 °C and 14,000*g* for 10 min for analysis.

## Sample detections

### UPLC–ESI–Q–TOF–MS/MS analysis

An AB Sciex UPLC Exionl system (Waters Co., Milford, MA, USA) coupled with a triple time-of-flight (TOF) 5600^+^ mass spectrometer (Sciex-Foster, Redwood City, CA, USA) was employed for UPLC–triple–TOF/MS analysis in this study. A Waters Ethylene Bridged Hybrid (BEH) C_18_ column (1.7 µm, 2.1 mm × 100 mm; Waters Co., Milford, MA, USA) and an ACQUITYTM UPLC BEH C_18_ (1.7 µm, 5 mm × 2.1 mm; Waters Co., Milford, MA, USA) were used. The column temperature was maintained at 35 °C, and the autosampler temperature at 10 °C. A linear gradient elution was performed with 0.1% formic acid–water as mobile phase A and 0.1% formic acid–methanol as mobile phase B. The gradient elution program was as follows: 95–50% eluent A for 0–10 min, 50–30% eluent A for 10–13 min, 30% eluent A for 13–15 min, 30–0% eluent A for 15–20 min, 0–95% eluent A for 20–20.1 min, and 95% eluent A for 20.1–25 min. The flow rate was set to 0.3 mL/min, and the injection volume was 3 μL. The mass spectrometer was operated in negative and positive ion modes. All the samples were kept at 4 °C during the analysis.

### Mass spectrometry

Electrospray ionization (ESI) mass spectrometry was used in separate injections for positive and negative ion modes with dynamic background subtraction. The ESI source operation conditions were as follows. In positive electrospray, the ion source voltage was set at 5,500 V, source temperature at 550 °C, nebulizer gas (GS1) at 55 psi, auxiliary gas (GS2) at 55 psi, curtain gas (CUR) at 35 psi, declustering potential (DP) at 80 V, collision energy (CE) at 35 V, and the collision energy spread (CES) at 15 EV. TOF–MS was operated in full scan at *m/z* 100–1,500 Da, and the information-dependent acquisition (IDA) mode was applied, with eight most intense peaks exceeding 100 cps for MS/MS scans and the production ion set at *m/z* 50–1,500 Da. In negative electrospray, the ion source voltage was set at −4,500 V, source temperature at 550 °C, GS1 at 55 psi, GS2 at 55 psi, CUR at 35 psi, DP at 80 V, CE at 35 V, and CES at 15 EV. TOF–MS was operated in full scan at *m/z* 100–1,500 Da, and the IDA mode was applied, with eight most intense peaks exceeding 100 cps for MS/MS scans and the product ion at *m/z* 50–1,500 Da. AnalystTF (version 1.6; AB SCIEX, Framingham, MA, USA) software was used for data acquisition. Data analysis and processing were performed using PeakView 2.0 software and Master View 1.0 (AB Sceix, Framingham, MA, USA).

### Data analysis

The metabolomics analysis was performed on UPLC-Q-TOF/MS data, and the mass number, abundance, and secondary mass of the chromatographic peaks were obtained. The PeakView 2.0 software with Formula Finder (AB Sceix, Framingham, MA, USA) was used to estimate the chemical composition. The total ion chromatogram was imported to PeakView2.0 (AB Sceix, Framingham, MA, USA) to elucidate the compounds and determine the exact mass. The molecular formula of the chromatographic peaks of the *S. divaricata* samples was determined following the principle of deviations of the experimental values from the theoretical values below 5 ppm and the fit of abundance less than 1.0, combined with the fragment of secondary mass spectrometry, mass spectrometry fragment of standards, and the previous findings. The molecular composition and structure that could not be obtained from the MS/MS fragmentation and chromatography behavior, were inferred by referring to ChemSpider databases (www.chemspider.com).

The resulting peak list was further processed using Microsoft Excel and imported into the SIMCA-P^+^ software (version 14.1; Umetrics; Sartorius AG, Göttingen, Germany) for orthogonal projections to latent structures discriminant analysis (OPLS-DA). OPLS-DA was performed using the scores plot for the compounds with large effects in the treatment groups to observe the differences in chemical components. The differential compounds were identified by comparing the mass-to-charge ratio and retention time. The target ions with high intergroup dispersion (variable influence on projection, VIP) >2 were collected, combined with the result of the intergroup *t-*test (*P* < 0.05) to identify the differential compounds.

### Determination of antioxidant enzyme activities and levels of O_2_^−.^ and MDA

All analyses were performed using kits (Nanjing Jiancheng Bioengineering Institute, Nanjing, China). The operating instructions were downloaded from at http://www.njjcbio.com/. The absorbance values of SOD, CAT, POD, phenylalanine ammonia-lyase (PAL) O_2_^.−^, and MDA were measured at 550, 405, 420, 290, 550 and 532 nm using an ultraviolet–visible spectrophotometer (UV-1600; Shimadzu, Kyoto, Japan), respectively. All operations and calculations followed the manufacturer’s protocol.

## Results

### Identification of metabolites

**Figure 1:**
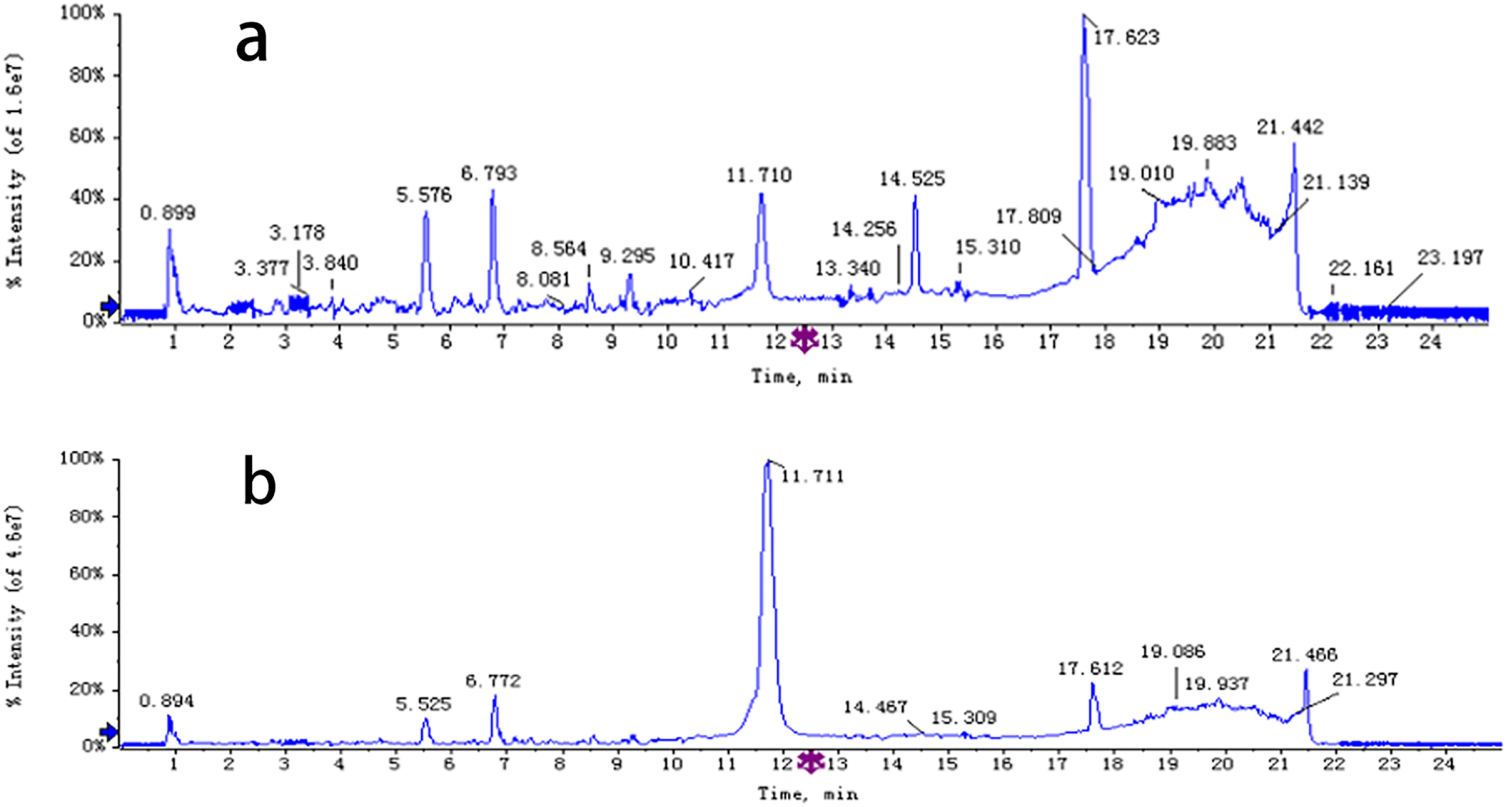
UPLC Chromatograms of *S*. *divaricata*. Analysed in positive ionization mode and negative ionization mode. (A) positive ion mode; (B) negative ion mode.

**Figure 2:**
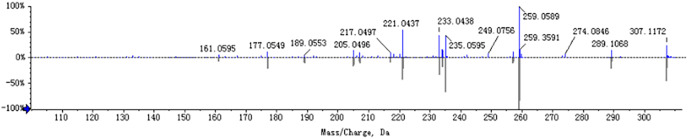
MS/MS spectra (ESI^+^) of cimifugin. The mirror image is the MS/MS fragment of cimifugin standard.

**Figure 3:**
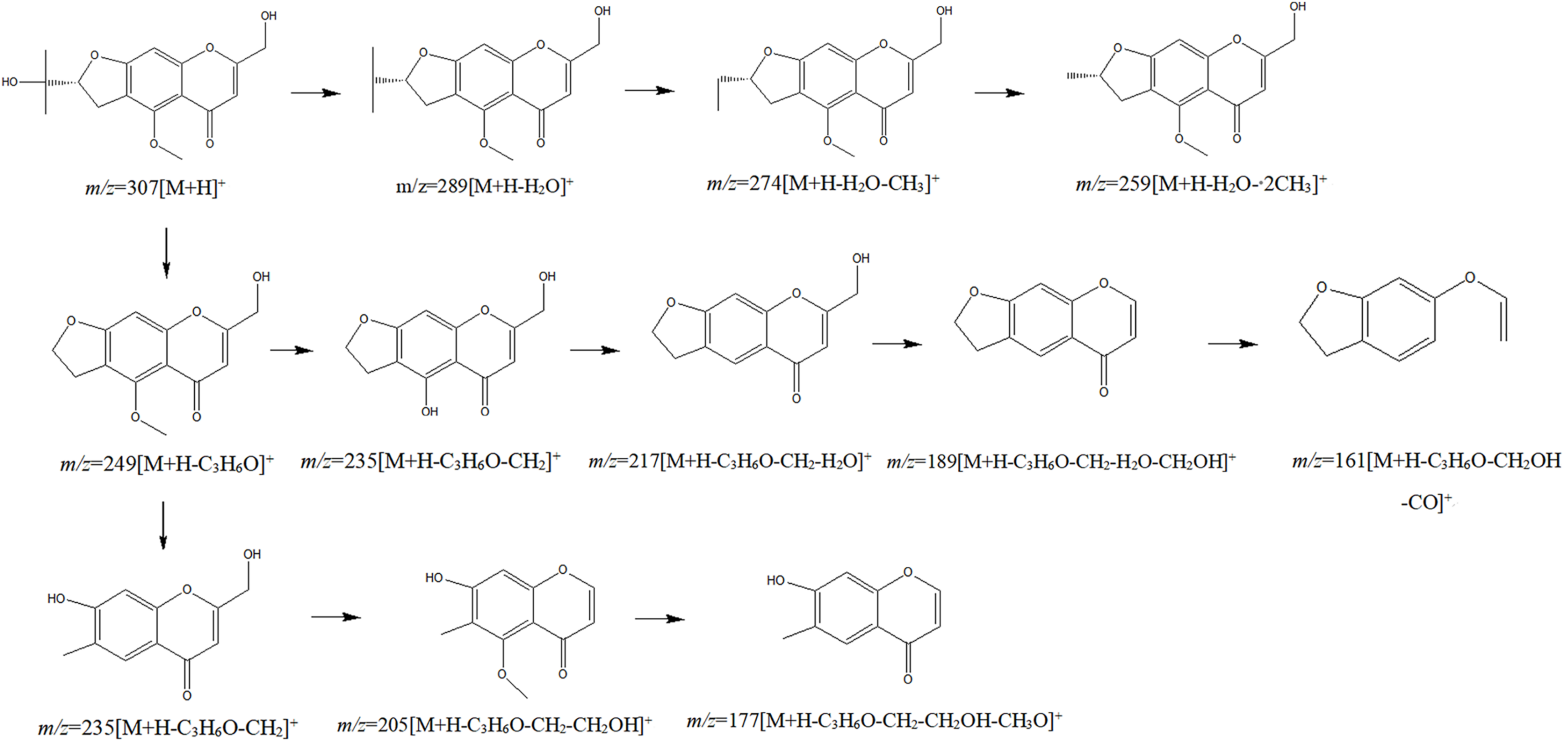
Splitting decomposition law inference (ESI^+^) of cimifugin based on UPLC-MS analysis.

**Figure 4:**
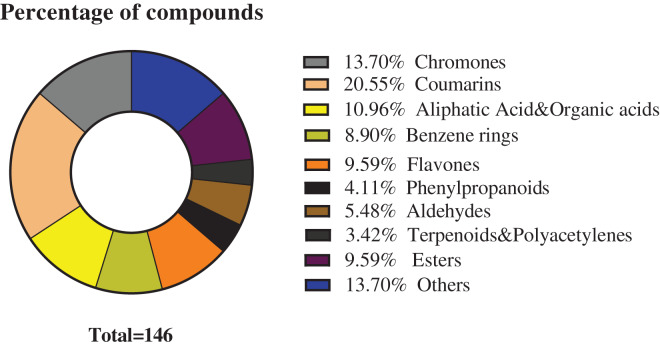
Classification of the 146 metabolites of *S*. *divaricata*.

The chemical composition of *S. divaricata* was elucidated *via* the secondary ion mass spectrometry (Fig. 1 UPLC Chromatograms of *S. divaricate*), in terms of the retention time, mass-to-charge ratio, molecular weight, structural formula, and elemental composition of known ingredients in *S. divaricata*. Cimifugin was used as a reference to identify the chemical composition. The ion (Retention Time = 6.43 min, [M + H]^+^ = 307.12) was detected as C_16_H_19_O_6_ in positive ion mode based on elemental composition, isotopic abundance fraction, and ChemSpider database from the *S. divaricata* roots. The MS/MS fragment ion of peak 13 at *m/z* 289 Da was identified as C_16_H_17_O_5_^−^, which the fragment formed after C_16_H_19_O_6_ dehydration; the MS/MS fragment ion of peak 12 at *m/z* 274 Da was C_15_H_14_O_5_^−^ obtained from C_16_H_17_O_5_^−^ after CH_3_ removal; and the MS/MS ion at *m/z* 249 Da of peak 9 was C_16_H_19_O_6_ formed after C_3_H_5_OH (58 Da) removal. These observations under different treatment groups confirmed the ion as cimifugin. The secondary ion mass spectrometry of cimifugin is shown in Fig. 2, and the ion cracking analysis process of cimifugin is shown in Fig. 3. Furthermore, 146 compounds, including 20 chromones, 30 coumarins, 16 aliphatic acids and organic acids, 13 benzene rings, 14 flavonoids, six phenylpropanoids, eight aldehydes, five terpenoids and polyacetylenes, 14 esters, and 20 other compounds, were identified from the fresh roots of *S. divaricata* based on the aforementioned analyses and the previous findings (Xue et al., 2019; Zhang et al., 2008; Li et al., 2006; Ma et al., 2018; Sun et al., 2022; Yang et al., 2015; Chen et al., 2018; Zhao et al., 2010; Yokosuka et al., 2017; Kreiner et al., 2017; Yoshitomi et al., 2020; Batsukh et al., 2020). The distribution of compounds is shown in Fig. 4.

### Differential compounds identified based on metabolomics analysis

**Figure 5:**
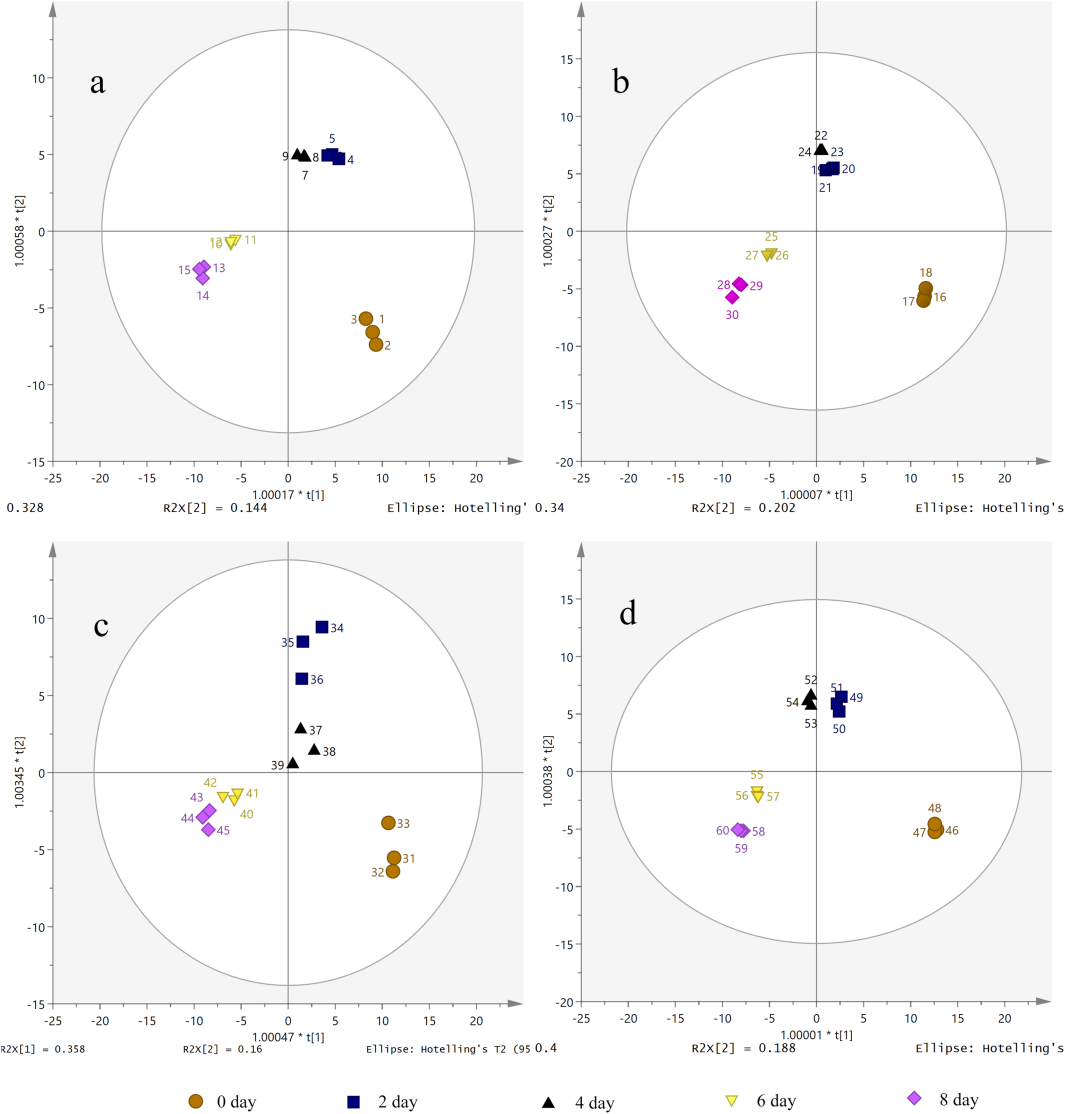
Orthogonal principal component analysis (OPLS-DA) score plots of metabolites under drought treatment. (A) CK treatment group; (B) 10% PEG-6000 treatment group; (C) 15% PEG-6000 treatment group; (D) 30% PEG-6000 treatment group. The brown (circle), dark blue (box), black (triangle), yellow (inverted triangle) and pink (diamond) points represent 0, 2, 4, 6, 8 days treatment respectively. Data are presented as three replicates.

The candidate ions of *S. divaricata* under different treatment durations and PEG-6000 concentrations were preliminarily determined according to OPLS-DA. The OPLS-DA score plots showed that the different drought treatment groups were clearly separated. As shown in Fig. 5, the different treatment groups were divided into five regions. Also, the repeated samples were placed together, indicating the significant chemical differences between the treatments. Therefore, the established metabolomics method was used to present the chemical characteristics successfully. The target ions with high intergroup dispersion (VIP > 2) were identified as the differential compounds. Significant changes were detected in 7 PMs and 28 SMs in the *S. divaricata* roots obtained from plants under drought stress, as shown in Table S1.

### Metabolomics data analysis of characteristic differential compounds

**Figure 6:**
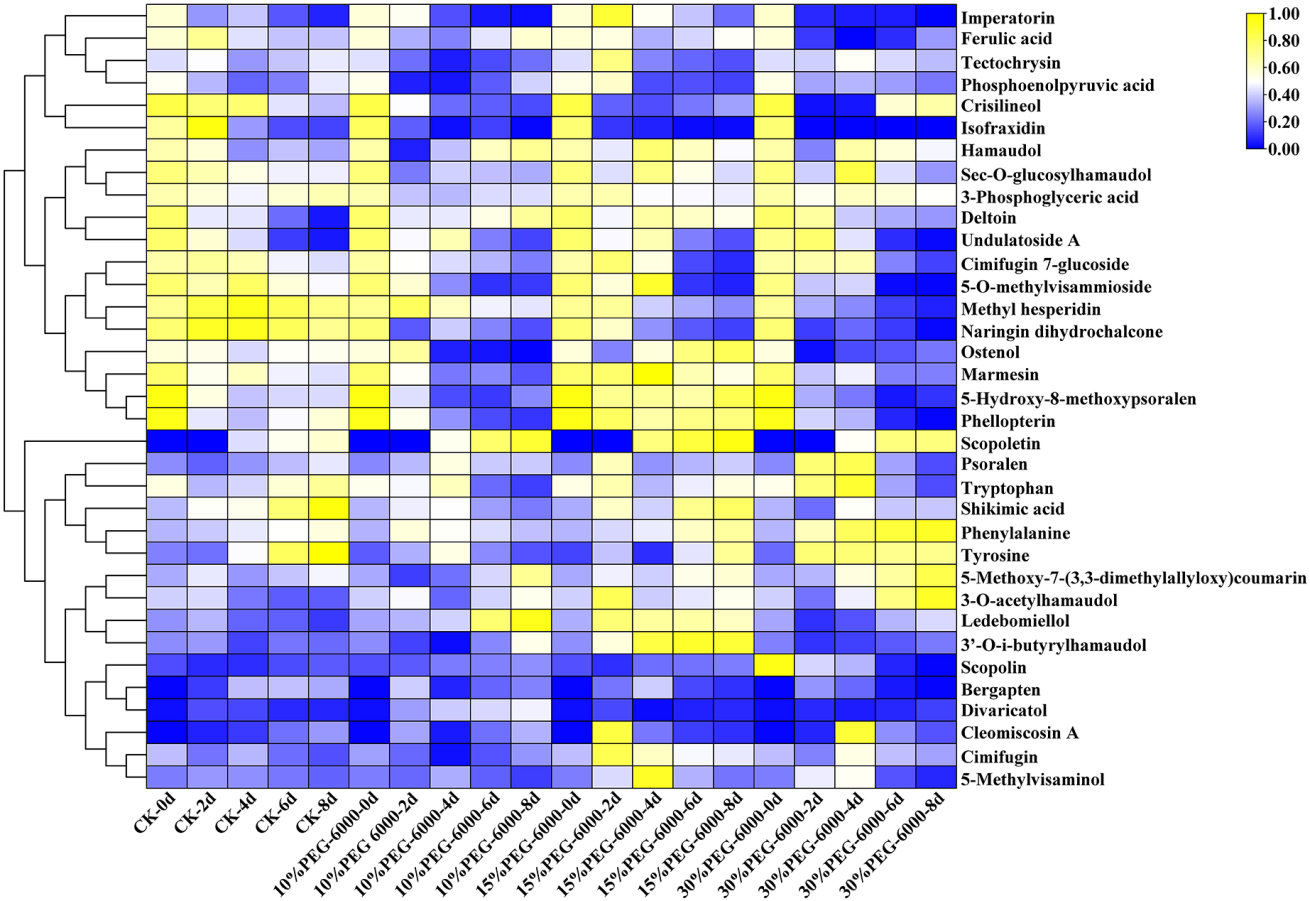
Heat map visualization of *S. divaricata* in different treatment groups under drought stress. The color range from blue to yellow indicates relative abundance from low to high (Color scale key above heat map). Data are presented as three replicates.

The hierarchical cluster analysis was performed using R software (www.r-project.org/). The metabolite content data were standardized, and the differences in cumulative metabolites between different samples were analyzed and displayed. As shown in Fig. 6, the differential PMs were 3-phosphoglyceric acid, phosphoenolpyruvic acid, tryptophan, shikimic acid, phenylalanine, Citric acid, and tyrosine. Citric acid was identified in the control group but not in the other three groups. Compared with the control group, the treatment groups showed a decrease in the levels of 3-phosphoglyceric acid, tryptophan, phosphoenolpyruvic acid, tyrosine, and shikimic acid and an increase in the level of phenylalanine. Meanwhile, the differential SMs were cimifugin, cimifugin 7-glucoside, hamaudol, sec-*O*-glucosylhamaudol, 3′-*O*-i-butyrylhamaudol, 3-*O*-acetylhamaudol, 5-*O*-methylvisammioside, 5-*O*-methylvisaminol, divaricatol, ledebouriellol, methyl hesperidin, tectochrysin, naringin dihydrochalcone, scopoletin, scopolin, deltoin, imperatorin, isofraxidin, phellopterin, crisilineol, marmesin, psoralen, bergapten, ostenol, 5-hydroxy-8-methoxypsoralen, 5-methoxy-7-(3,3-dimethylallyloxy)coumarin, cleomiscosin A, and ferulic acid. The treatment groups showed an increase in the levels of cimifugin, hamaudol, 3′-*O*-i-butyrylhamaudol, 3-*O*-acetylhamaudol, divaricatol, 5-*O*-methylvisaminol, ledebouriellol, scopoletin, imperatorin, phellopterin, deltoin, marmesin, psoralen, ostenol, 5-hydroxy-8-methoxypsoralen, 5-methoxy-7-(3,3-dimethylallyloxy) coumarin, ferulic acid, and scopolin and a decrease in cimifugin 7-glucoside, sec-*O*-glucosylhamaudol, 5-*O*-methylvisammioside, methyl hesperidin, cleomiscosin A, tectochrysin, naringin dihydrochalcone, isofraxidin, crisilineol, and bergapten compared with those in the group (Fig. 6).

### Antioxidant enzyme activities and the contents of superoxide anion (O_2_^−.^) and MDA

**Figure 7:**
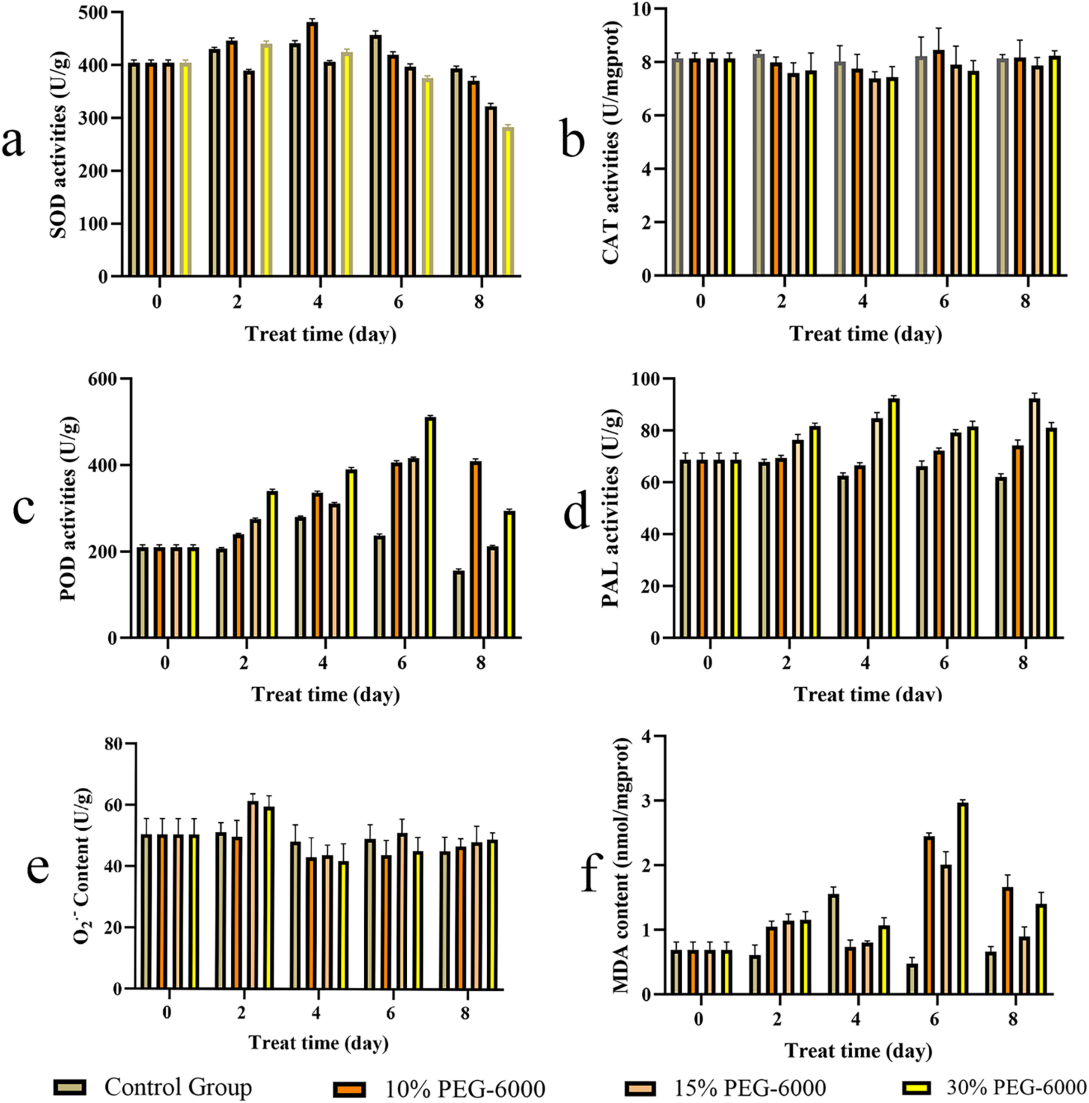
The contents of *S. divaricata* on antioxidant enzymes activities, superoxide anion (O_2_^.−^) and malondialdehyde (MDA) under drought stress. (A) Superoxide dismutase (SOD), (B) catalase (CAT), (C) peroxidase (POD), (D) phenylalanine ammonia lyase (PAL), (E) superoxide anion (O_2_^−.^) and (F) malondialdehyde (MDA). The brown, orange, light orange and yellow represent CK, 10%, 15% and 30% PEG-6000 treatment groups represently. Data are presented as three replicates.

The activities of SOD, CAT, POD, and PAL are shown in Fig. 7. The SOD activity in *S. divaricata* in the drought treatment groups was generally lower than that in the control group; SOD under drought showed an increase and then a decrease, as shown in Fig. 7A. The SOD activity increased significantly on day 4 of 10% PEG-6000 treatment and decreased on day 4–8 in each treatment group. The CAT activity in the treatment groups was generally lower than in the control group; The CAT under drought first reduced and then increased under drought conditions, as shown in Fig. 7B. The POD activity in the drought treatment groups was significantly higher than that in the control group, with the most significant change under severe drought conditions (Fig. 7C). The CAT activity under 10%, 15%, and 30% PEG-6000 treatment was 263.16%, 136.22%, and 188.63% higher than that in the control group, respectively, on the day 8. The PAL activity in each drought treatment group was higher than that in the control group; the enzyme activity first increased and then decreased, as shown in Fig. 7D. The PAL activity in the 15% and 30% PEG-6000 treatment groups was 135.68% and 147.74% higher than that in the control group, respectively, on day 4.

The content of O_2_^.−^ in *S. divaricata* represents the degree of damage to plant cells under drought conditions, which increased significantly in the 15% and 30% PEG-6000 treatment groups on day 2 (Fig. 7E). The MDA content represents the lipid peroxidation level of fresh roots under oxidative stress and indirectly reflects the damage level of cells. The MDA content in the drought treatment groups was close to that in the control group in the early stage of treatment, but increased significantly in the late stage (Fig. 7F). The MDA content increased significantly on day 6 and decreased on day 8 in each group, which was higher than that in the control group.

## Discussion

ROS are inevitably produced in plants for growth and development. Under mild drought, high temperature, and other adverse conditions, ROS at appropriate concentrations act as signal molecules and induce a series of physiological changes and adjustments in metabolism (Miller et al., 2010). However, when the plants are exposed to severe adversity for a long period, the scavenging system fails to eliminate the additional ROS generated and results in oxidative damage (Pallavi et al., 2012; Das, Majumder & Biswas, 2021). Drought is the main abiotic stress of plants, which leads to oxidative stress by producing different forms of ROS. A balance in ROS depends on their production and elimination, which is determined by the type, content, and activity of antioxidants (Qi et al., 2017).

### Drought promoted the transformation glycosides into aglycones

Glycoside molecules contain a hydrophilic glucose group without the ability to pass through biofilms. However, the glycosides are often enclosed in the cells with biofilms, and their antioxidant activity is reduced. As shown in Fig. 7E, the content of O_2_^−.^ increased significantly in the early stage and decreased in the following days under drought stress. However, plants have SMs besides antioxidant enzymes, which can convert the stored glycosides into aglycones to enhance the antioxidant effect and reduce the ROS produced under oxidative stress. As shown in Fig. 6, most of the 28 SMs detected under drought stress were glycosides and their glycosidic counterparts, such as cimifugin and cimifugin 7-glucoside, 5-*O*-methylvisammioside and 5-*O*-methylvisaminol, hamaudol and sec-*O*-glucosylhamaudol, scopoletin and scopolin, and marmesin, which got interchanged under the action of hydrolases and glucosaminidases. This results in the accumulation of compounds with stronger antioxidant activity, which was consistent with researches of the previous findings (Shen et al., 2020; Karuppusamy, 2010; Ahmed et al., 2021; Martim, 2014). The model plant used in present study, *S. divaricata*, is a common medicinal plant; cimifugin with excellent analgesic, antipyretic, and anti-inflammatory effects is the main component of this plant (State Pharmacopoeia Committee, 2020; Yang et al., 2017; Lee et al., 2020; Wang et al., 2017). Cimifugin 7-glucoside and 5-*O*-methylvisammioside have little pharmacodynamic action unless the hydrophilic sugar molecules are removed and converted into the corresponding nonglycosidic components *in vivo* (Han et al., 2017). Under favorable conditions, the intracellular ROS are present at moderate concentrations, and the glycoside components, such as cimifugin 7-glucoside, 5-*O*-methylvisammioside, hamaudol, and scopolin, are stored at high levels but with low activities. However, under adverse conditions, cimifugin 7-glucoside, 5-*O*-methylvisammioside, hamaudol, imperatorin, and scopolin quickly remove a glucose molecule and get converted into cimifugin, sec-*O*-glucosylhamaudol, 5-*O*-methylvisaminol, and scopoletin under the action of the hydrolytic enzymes (Chen et al., 2018; Zhao et al., 2016; Döll et al., 2018), which is consistent with the results of the present study shown in Fig. 6. The antioxidant effects are enhanced due to the additional –OH group after removing the sugar base by hydrolysis. Therefore, the levels of cimifugin 7-glucoside, 5-*O*-methylvisammioside, and sec-*O*-glucosylhamaudol decreased rapidly under oxidative stress, while the levels of cimifugin 5-*O*-methylvisaminol, and hamaudol increased. Hence, the number of –OH groups in metabolites increased, and the antioxidant activity was enhanced accordingly. When the ROS level decreased in a suitable environment, excessive free aglycones of cimifugin, 5-*O*-methylvisaminol, and hamaudol were converted into the bound state by glucosyltransferases, leading to the decreased activities. These observations indicated that the activities of glycosides and their glycosidic counterparts varied greatly with the environment changes. The conversion of glycosides and their corresponding products needed only one-step hydrolysis and biosynthesis, which could quickly complete the reaction in cells. Therefore, it can quickly adjust the antioxidant capacity and adapt to the environment. Thus, glycosides and their glycosidic counterparts acted as a “buffer pair” maintaining the redox balance and stabilizing the ROS level rapidly in cells when the external environment changed.

### Drought promoted active metabolite generation

**Figure 8:**
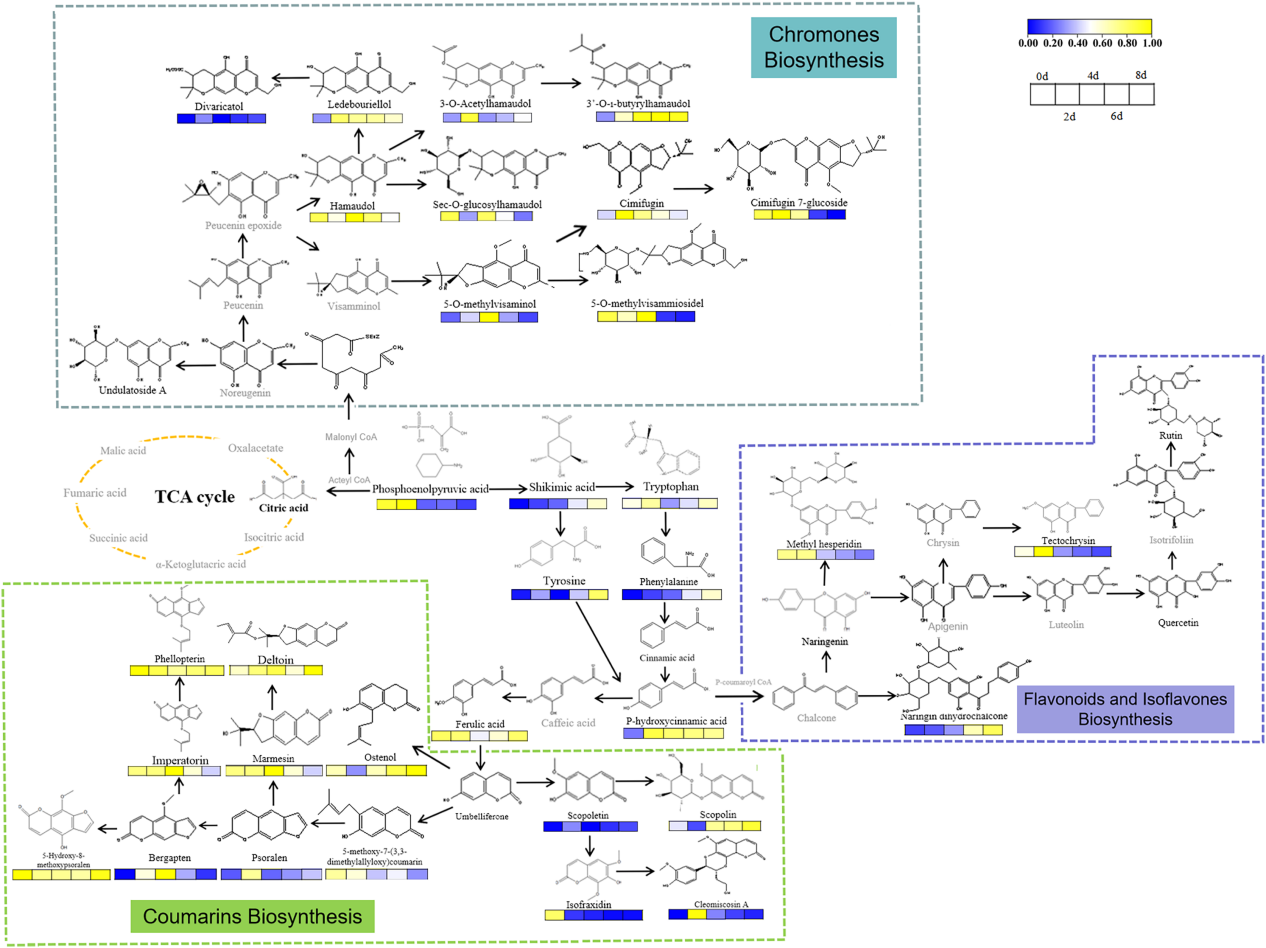
Metabolic networks of *S. divaricata* under drought stress. The proposed metabolic pathways were based on the current research and web-based database of metabolic pathways. Content changes of difference compounds were presented by color changes. Metabolites in gray are not found in this study. The changes of metabolite content were based on the drought stress data stimulated by 15% PEG-6000, and the changes of metabolite content at different treatment times were compared based on the control. The content change trend has been indicated in the figure. Metabolites in blue represent decrease whereas yellow represent increase.

Drought induced changes in levels of PMs in the fresh roots of *S. divaricata*. Citric acid, which is an important component of the tricarboxylic acid (TCA) cycle, was detected in the control group but not in the other three drought treatment groups, indicating that the oxidative stress inhibited the primary metabolism in *S. divaricata*. As the intermediate substances in transforming PMs into secondary metabolites (Pant, Pandey & Dall’Acqua, 2021), the levels of tryptophan, tyrosine, and shikimic acid increased during the treatment, indicating that oxidative stress enhanced the biosynthesis of SMs (Qian et al., 2019; Guan et al., 2021; Yokoyama et al., 2021). The increase in the levels of downstream substances, such as 5-*O*-methylvisaminol, inevitably required raw materials obtained from 3-phosphoglyceric acid and phosphoenolpyruvic acid, which were the products of glycolysis (Pacold & Anderson, 1973). Although these compounds were continuously consumed, their levels still showed a slight increase, indicating the promotion of glycolysis under oxidative stress. The synthetic pathway of *S. divaricata* under drought stress is shown in Fig. 8. These observations suggested that severe oxidative stress induced SMs *via* a negative feedback pathway, indicating a increased adaptability of *S. divaricata*.

### Drought enhanced the levels of highly SMs

Drought enhanced the levels of SMs in *S. divaricata* metabolites such as imperatorin, scopoletin, psoralen, cleomiscosin A, 5-hydroxy-8-methoxypsoralen, cimifugin, hamaudol, 5-*O*-methylvisamitol, bergapten, and 5-methoxy-7-(3,3-dimethylallyloxy)coumarin, which were first synthesized rapidly. These upstream substances, which lacked sugar groups and had low molecular weight, could diffuse freely into the cells but were synthesized rapidly, improving the antioxidant effect. More importantly, the amount and the kinds of active groups in the molecules determined the activities of chemicals (Wang et al., 2020b; Kawaii, Ishikawa & Yoshizawa, 2018). Moreover, these upstream substances had more –OH, –OCH_3_, and unsaturated double bonds, which directly determined the antioxidant ability of cells (Naikoo et al., 2019; Zheng et al., 2017; Avila-Nava et al., 2021). Once the level of ROS was reduced to an appropriate level, these upstream, high-activity products were converted into downstream, low-activity products and stored. The results indicated that the secondary metabolites can protect against oxidative stress.

### Regulatory roles of antioxidases

Drought induces various reactions, including oxidative stress, growth inhibition and synthesis of some nontoxic compounds, to increase the osmotic potential of plant cells and thus allow metabolic processes to enhance the activities of some antioxidant enzymes. Previous studies showed that the changes in SOD activities depended on the severity, duration, and type of drought (Quartacci & Navari-Izzo, 1992; Badiani et al., 1990). SOD, which can catalyze the conversion of O_2_^−.^ into H_2_O_2_, widely exists in plants and participates in almost all metabolic processes. In the present study, the antioxidation of *S. divaricata* under early or mild drought stress increased (Fig. 7A). However, SOD could not completely eliminate ROS, while H_2_O_2_ was mainly eliminated by CAT and POD (Hao et al., 2019; Jiang & Zhang, 2002; Dhanda, Sethi & Behl, 2010). SOD activity was lower under moderate and severe drought conditions than that of control group, indicating a limited oxidative stress resistance effect of SOD. Thus, SOD is not a key enzyme in the antioxidant enzyme system of *S. divaricata* under aggravated or long-term oxidative stress. The activity of CAT in each drought treatment group was similar to that of the control group (Fig. 7B), indicating that the drought conditions induced by PEG-6000 had little effect on CAT, which was consistent with the previous findings (Fuchino et al., 2021; Fu & Huang, 2001). Meanwhile, POD can effectively remove H_2_O_2_ produced when SOD scavenges O_2_^−.^ (Liu et al., 2014; Khan et al., 2021). The results showed that the activity of POD increased continuously in the low-concentration group and showed an increase at first and then a decrease at medium and high concentrations (Fig. 7C), indicating that POD scavenged ROS under oxidative stress, which was consistent with previous findings on different plants (Bano et al., 2021; Jing et al., 2013; Rangani et al., 2018; Ayyaz et al., 2021). In this study, the content of O_2_^−.^ increased significantly on day 2 under 15% and 30% PEG-6000 treatments and decreased in the following days (Fig. 7E). These changes were highly correlated with the high activities of SOD, POD, and CAT (Figs. 7A–7C), which effectively eliminated ROS in the early and middle stages of treatment and maintained a dynamic balance between the production and elimination of ROS, these observations were consistent with previous findings (Fu & Huang, 2001). However, the protective effect of antioxidant enzymes on plants was reduced in the late stage of drought, resulting in a large accumulation of ROS, which were transformed into more active ·OH *via* Haber–Weiss and Fenton reactions (Zorov, Juhaszova & Sollott, 2014). These changes intensified membrane lipid peroxidation and significantly increased MDA content. However, the antioxidant enzymes are also proteins, and their antioxidant activity is reduced under a long-term adverse environment. Therefore, other pathways are needed to exert synergistic antioxidant effects. The present study showed that the main components of *S. divaricata* were chromones, coumarins, and flavones derived *via* the phenylalanine biosynthesis pathway. PAL is the rate-limiting enzyme of the phenylalanine biosynthesis pathway, which is closely related to the generation of SMs in *S. divaricata* (Parra-Galindo et al., 2021; Li et al., 2013; Yao et al., 2017). In this study, the moderate and severe drought stresses improved the PAL activities in *S. divaricata* (Fig. 7D), showing that oxidative stress increased the rapid production of phenylalanine biosynthetic pathway compounds in *S. divaricata*, which was consistent with the change displayed in Fig. 6.

The activities of compounds depend on their active groups, such as –OH, –CH_3_, and double bonds (Naikoo et al., 2019; Zheng et al., 2017; Avila-Nava et al., 2021). Generally, phenolics with more –OH are considered the most important group of SMs, and the activity of –OH is much higher than that of –OCH_3_. However, the major SMs in *S. divaricata* were chromones with –OCH_3_ and not –OH; the chemical composition indicated weak adaptability for *S. divaricata*. Meanwhile, O^−.^_2_ gets converted into H_2_O_2_ mainly under the action of CAT or POD. However, POD in plants is a glycosylated protein, and the glycosylation can stabilize the conformation of enzymes and avoid protease degradation. Therefore, POD shows better stability and is the main enzyme for scavenging ROS under adverse conditions (Han et al., 2017; Qi et al., 2016). Numerous studies have shown that POD is an environment-induced enzyme. Its biosynthesis and activity increase rapidly under adverse conditions. However, unlike CAT and POD, *S. divaricata* is rich in chromones, and no hydrogen donors are required to scavenge ROS. Therefore, POD regulated the activities of SMs in *S. divaricata*, while the environment regulated the POD activity in this study. Under environmental stress, chromones can play a better role in antioxidation.

## Conclusion

The present study found that the SMs played essential roles in regulating the adaptability of *S. divaricata* to adversity. The plant *S. divaricata* regulates the content and activity of various chemical compounds *via* the biosynthesis of SMs and the transformation of different active ingredients to protect against oxidative stress. The content and activity of free SMs with phenolic hydroxyl and high-antioxidant activity groups increase under oxidative stress, and excess ROS are scavenged. These substances can be transformed into less-active substances and stored under favorable conditions. The SMs in *S. divaricata* are numerous, just like the “buffer pair” of chemical solutions, which maintain ROS balance to adapt to changing environments. Thus, the present study confirmed that the quality of herbal medicine was closely related to the SMs, which explained the diversity and complexity and the effect of moderate ecological stress in medicinal plants.

## Supplemental Information

10.7717/peerj.14336/supp-1 Supplemental Information 1 Differential metabolites in PEG-6000 sitmulated *S*. *divaricata*.

10.7717/peerj.14336/supp-2 Supplemental Information 2 Classification of 146 metabolites based on *Saposhnikovia divaricata*.

## Additional Information and Declarations

### Field Study Permissions

The following information was supplied relating to field study approvals (*i.e*., approving body and any reference numbers): Field experiments were approved by Heilongjiang College of traditional Chinese Medicine (20210113).

### Data Availability

The following information was supplied regarding data availability: The UPLC-MS data is available in Figshare: Cao, Sisi; Shi, Lei; Shen, Ying; Meng, Xiangcai (2022): Secondary metabolites of *Saposhnikovia divaricata* regulate adaptation to drought stress. figshare. Dataset. https://doi.org/10.6084/m9.figshare.20115731.v1.
