# Supplementary material for: Ecological roles of secondary metabolites of Saposhnikovia divaricata in adaptation to drought stress
Source: PeerJ. 2022 Nov 4;10:e14336. doi: 10.7717/peerj.14336 (PMC9639429; doi:10.7717/peerj.14336)
Supplement: Supplemental Information 1 [file peerj-10-14336-s001.docx]

**Table 1 Differential metabolites in PEG-6000 sitmulated *S. divaricata***

| **Peak**  **No.** | **Rt**  **(min)** | **Selected ion** | **Measured mass (*m/z*)** | **Calc. Mass\**  **(*m/z*)** | **Error**  **(ppm)** | **Formula** | **MS/MS fragment ion （*m/z*)** | **Identification** |
| --- | --- | --- | --- | --- | --- | --- | --- | --- |
| 1 | 0.81 | [M+H]^+^ | 175.11941 | 175.11745 | -0.3 | C_7_H_10_O_5_ | 175M+H]^+^, 158[M+H-OH^]+^, 130[M+H-OH-CO]^+^ | shikimic acid |
| 2 | 0.93 | [M+H]^+^ | 249.11483 | 249.11214 | 5.8 | C_14_H_16_O_4_ | 249[M+H]^+^, 233[M+H-CH_3_]^+^, 231[M+H-H_2_O]^+^, 203[M+H-CO-H_2_O]^+^, 189[M+H-CH_3_-CO_2_]^+^, 185[M+H-2CO-H_2_O]^+^, 174[M+H-2CH_3_-CO_2_]^+^, 171[M+H-CH_3_-CO_2_-H_2_O]^+^, 161[M+H-CH_3_-CO_2_-CO]^+^ | 5-methoxy-7-(3,3-dimethylallyloxy)coumarin |
| 3 | 1.52 | [M+H]^+^ | 182.08153 | 182.08225 | 5.2 | C_9_H_11_NO_3_ | 182[M+H]^+^, 165[M+H-OH]^+^, 148[M+H-2OH]^+^, 120[M+H-2OH-CO]^+^ | tyrosine |
| 4 | 1.54 | [M+H]^+^ | 165.05455 | 165.05441 | -1.5 | C_20_H_26_O_6_ | 165[M+H]^+^, 148[M+H-OH]^+^, 120[M+H-OH-CO]^+^, 131[M+H-2OH]^+^ | p-hydroxycinnamic acid |
| 5 | 2.67 | [M+H]^+^ | 164.08711 | 164.08676 | -1.5 | C_9_H_11_NO_2_ | 164[M+H]^+^, 147[M+H-OH]^+^, 119[M+H-OH-CO]^+^, 148[M+H-NH2]^+^ | phenylalanine |
| 6 | 3.68 | [M+H]^+^ | 205.09711 | 205.09715 | -0.7 | C_11_H_12_N_2_O_2_ | 205[M+H]^+^, 188[M+H-OH]^+^, 160[M+H-OH-CO]^+^, 144[M+H-OH-CO-NH_2_]^+^ | tryptophan |
| 7 | 5.32 | [M+H]^+^ | 469.16760 | 469.17044 | -4.1 | C_22_H_28_O_11_ | 469[M+H]^+^, 451[M+H-H_2_O]^+^, 397[M+H-C_3_H_8_O]^+^, 307[M+H-C_6_H_10_O_5_]^+^, 290[M+H-C_6_H_10_O_5_-H_2_O]^+^, 261[M+H-C_6_H1_0_O_5_-H_2_O-CH_2_O]^+^, 235[M+H-C_3_H_8_O-C_6_H_10_O_5_]^+^, 221[M+H-C_3_H_8_O-C_6_H_10_O_5_-CH_2_]^+^ | cimifugin 7-glucoside |
| 8 | 5.40 | [M+H]^+^ | 193.04901 | 193.04954 | -2.7 | C_10_H_8_O_4_ | 193[M+H]^+^, 178[M+H-CH_3_]^+^, 161[M+H-CH_3_-OH]^+^, 133[M+H-CH_3_-OH-CO]^+^, 165[M+H-CO]^+^, 137[M+H-2CO]^+^, 149[M+H-CO_2_]^+^, 50[M+H-CH_3_-CO]^+^, 122[M+H-CH_3_-2CO]+, 105[M+H-CH_3_-2CO-OH]^+^ | scopoletin |
| 9 | 5.60 | [M+H]^+^ | 345.09550 | 345.09688 | -4.0 | C_18_H_16_O_7_ | 345[M+H]^+^, 326[M+H-H_2_O]^+^, 309[M+H-2H_2_O]^+^, 281[M+H-2H_2_O -CO]^+^, 255[M+H-H_2_O-CO_2_-CO]^+^ | crisilineol |
| 10 | 5.80 | [M-H]^-^ | 195.06493 | 195.06519 | -1.5 | C_10_H_11_O_4_ | 195[M-H]^-^, 177[M-H-H_2_O]^-^, 149[M-H-H_2_O-CO]^-^, 134[M-H-H_2_O-CO-CH_3_]^-^, 135[M-H- H_2_O-CO-CH_2_]^-^, 117[M-H-2H_2_O-CO-CH_2_]^-^ | ferulic acid |
| 11 | 0. | [M+H]- | 193.05011 | 193.05140 | -0.5 | C₆H₈O₇ | 343[M+H]^+^, 325[M+H-H_2_O]^+^, 307[M+H-2H_2_O]^+^, 293[M+H-2H_2_O-CH_2_]^+^, 265[M+H-2H_2_O-C_3_H_6_]^+^, 250[M+H-2H_2_O-C_4_H_9_]^+^ | citric acid |
| 12 | 6.39 | [M+H]^+^ | 247.09860 | 247.09649 | 4.5 | C_14_H_14_O_4_ | 247[M+H]^+^, 229[M+H-H_2_O]^+^, 214[M+H-CH_3_-H_2_O]^+^, 213[M+H-H_2_O-CH_4_]^+^, 211[M+H-2H_2_O]^+^, 201[M+H-H_2_O-CO]^+^, 175[M+H-4H_2_O]^+^, 147[M+H-4H_2_O-CO]^+^, 119[M+H-4H_2_O-2CO]^+^ | marmesin |
| 13 | 6.40 | [M+H]^+^ | 329.13954 | 329.13835 | 3.5 | C_19_H_20_O_5_ | 329[M+H]^+^, 247[M+H-C_5_H_6_O]^+^, 229[M+H-C_5_H_6_O-H_2_O]^+^, 213[M+H-C_5_H_6_O-H_2_O-CH_3_]^+^, 185[M+H-C_5_H_6_O-H_2_O-CO_2_]^+^, 175[M+H-C_5_H_6_O-H_2_O-C_4_H_6_]^+^ | deltoin |
| 14 | 6.43 | [M+H]^+^ | 307.11720 | 307.11762 | -1.4 | C_16_H_18_O_6_ | 307[M+H]^+^, 289[M+H-H_2_O]^+^, 274[M+H-H_2_O-CH_3_]^+^, 259[M+H-C_2_H_8_O]^+^, 257[M+H-H_2_O-CH_3_-OH]^+^, 249[M+H-C_3_H_6_O]^+^, 235[M+H-C_3_H_6_O-CH_2_]^+^, 233[M+H-C_3_H_6_O-CH_4_]^+^, 221[M+H-C_3_H_6_O-2CH_2_]^+^, 217[M+H-C_3_H_6_O-CH_2_-H_2_O]^+^, 205[M+H-C_3_H_6_O-CH_4_-CO]^+^, 189[M+H-C_3_H_6_O-CH_2_-H_2_O-CO]^+^, 177[M+H-C_3_H_6_O-CH_4_-2CO]^+^, 161[M+H-C_3_H_6_O-CH_2_-H_2_O-2CO]^+^ | cimifugin |
| 15 | 6.80 | [M+H]^+^ | 453.17552 | 453.17552 | 0 | C_22_H_28_O_10_ | 453[M+H]^+^, 291[M+H-glu]^+^, 273[M+H-GLU-H_2_O]^+^, 258[M+H-GLU-H_2_O-CH_3_]^+^, 245[M+H-GLU-H_2_O-CO]^+^, 243[M+H-GLU-H_2_O-C_2_H_6_]^+^, 231[M+H-GLU-H_2_O-CH_3_-C_3_H_6_]^+^, 216[M+H-GLU-H_2_O-CH_3_-2C_3_H_6_]^+^, 203[M+H-GLU-H_2_O-CH_3_-C_3_H_6_-CO]^+^ | 5-O-methylvisammioside |
| 16 | 7.10 | [M+H]^+^ | 625.21039 | 625.21270 | -3.7 | C_29_H_36_O_15_ | 625[M+H]^+^, 607[M+H-H_2_O]^+^ | methyl hesperidin |
| 17 | 7.20 | [M+H]^+^ | 269.08053 | 269.08084 | -3.2 | C_16_H_12_O_4_ | 269[M+H]^+^, 251[M+H-H_2_O]^+^, 223[M+H-H_2_O-CO]^+^, 213[M+H-2CO]^+^, 195[M+H-H_2_O-2CO]^+^ | tectochrysin |
| 18 | 7.30 | [M+H]^+^ | 291.12247 | 291.12276 | -1.0 | C_16_H_18_O_5_ | 291[M+H]^+^, 273[M+H-H_2_O]^+^, 233[M+H-H_2_O-C_3_H_4_]^+^, 205[M+H-H_2_O - C_3_H_4_-CO]^+^, 194[M+H- H_2_O-C_4_H_6_-CO]^+^ | 5-methylvisaminol |
| 19 | 7.50 | [M+H]^+^ | 439.15965 | 439.15987 | -0.6 | C_21_H_26_O_10_ | 439[M+H]^+^, 277[M+H-C_6_H_10_O_5_]^+^, 259[M+H-C_6_H_10_O_5_-H_2_O]^+^, 241[M+H-2H_2_O-C_6_H_10_O_5_]^+^, 217[M+H-C_6_H_10_O_5_-C_3_H_8_O]^+^, 189[M+H-C_6_H_10_O_5_-C_3_H_8_O-CO]^+^, 205[M+H-4H_2_O-C_6_H_10_O_5_]^+^ | sec-O-glucosylhamaudol |
| 20 | 7.90 | [M+H]^+^ | 387.10731 | 387.10744 | -0.3 | C_20_H_18_O_8_ | 387[M+H]^+^, 369[M+H-H_2_O]^+^, 357[M+H-2CH_3_]^+^ | cleomiscosin A |
| 21 | 8.50 | [M+H]^+^ | 187.03880 | 187.03897 | -5.2 | C_11_H_6_O_3_ | 187[M+H]^+^, 159[M+H-CO]^+^, 143[M+H-CO_2_]^+^, 131[M+H-2CO]^+^, 103[M+H-3CO]^+^ | psoralen |
| 22 | 8.56 | [M+H]^+^ | 277.10911 | 277.10705 | 4.4 | C_15_H_16_O_5_ | 277[M+H]^+^, 259[M+H-H_2_O]^+^, 244[M+H-H_2_O-CH_3_]^+^, 229[M+H-H_2_O-2CH3]^+^, 217[M+H-H_2_O-C_3_H_6_]^+^, 205[M+H-C_4_H_8_O_2_]^+^, 201[M+H-H_2_O-C_3_H_6_O]+, 189[M+H-H_2_O-C_3_H_6_-CO]^+^, 177[M+H-H_2_O-C_5_H_6_O]^+^, 165[M+H-H_2_O-C_3_H_6_-C_4_H_4_]^+^, 151[M+H-H_2_O-C_5_H_6_O-C_2_H_2_]^+^, 137[M+H-H_2_O-C_5_H_6_O-C_2_H_2_-CH_2_]^+^, 123[M+H-H_2_O-C_5_H_6_O-C_2_H_2_-2CH_2_]^+^ | hamaudol |
| 23 | 9.70 | [M+H]^+^ | 217.04890 | 217.04954 | -3.0 | C_12_H_8_O_4_ | 217[M+H]^+^, 202[M+H-CH_3_]^+^, 189[M+H-CO-CH_3_]^+^, 161[M+H-2CO-CH_3_]^+^, 174[M+H-CH_3_-CO]^+^, 146[M+H-CH_3_-2CO]^+^, 181[M+H-CH_3_-3CO]^+^ | bergapten |
| 24 | 9.80 | [M+H]^+^ | 335.11273 | 335.11253 | 0.6 | C_17_H_18_O_7_ | 335[M+H]^+^, 275[M+H-C_2_H_4_O_2_]^+^, 260[M+H-C_2_H_4_O_2_-CH_3_]^+^, 247[M+H-C_2_H_4_O_2_-CO]^+^, 233[M+H-C_2_H_4_O_2_-C_3_H_6_]^+^, 221[M+H-C_6_H_10_O_2_]^+^, 205[M+H-C_2_H_4_O_2_-C_3_H_6_-CO]^+^, 187[M+H-C_2_H_4_O_2_-C_3_H_6_-CO-H_2_O]^+^, 174[M+H-C_2_H_4_O_2_-CH_3_-CO_2_-CO-CH_2_]^+^, 177M+H-C_2_H_4_O_2_-C_3_H_6_-2CO]^+^, 159[M+H-C_2_H_4_O_2_-C_3_H_6_-2CO-H_2_O]^+^, 131[M+H-C_2_H_4_O_2_-C_3_H_6_-3CO-H_2_O]^+^ | divaricatol |
| 25 | 10.20 | [M+H]^+^ | 231.10125 | 231.10157 | -1.6 | C_14_H_14_O_3_ | 231[M+H]^+^, 189[M+H-C_3_H_6_]^+^, 175[M+H-C_4_H_8_]^+^, 147[M+H-C_4_H_8_-CO]^+^, 119[M+H-C_4_H_8_-2CO]^+^ | ostenol |
| 26 | 10.39 | [M+H]^+^ | 583.20287 | 583.20213 | 1.1 | C_27_H_34_O_14_ | 583[M+H]^+^, 551[M+H-CH_3_-OH]^+^, 539[M+H-CO_2_]^+^, 507[M+H-CO_2_-CH_3_OH]^+^, 489[M+H-CO_2_-CH_3_OH-H_2_O]^+^, 439[M+H-CO_2_-C_5_H_8_O_2_]^+^, 319[M+H-CO_2_-C_5_H_8_O_2_-4CH_2_O]^+^, 277[M+H-CO_2_-C_5_H_8_O_2_-4CH_2_O-C_2_H_2_O]^+^, 259[M+H-CO_2_-C_5_H_8_O_2_-4CH_2_O-C_2_H_2_O-H_2_O]^+^, 217[M+H-CO_2_-C_5_H_8_O_2_-4CH_2_O-C_2_H_2_O-H_2_O-C_3_H_6_]^+^, 205[M+H-CO_2_-C_5_H_8_O_2_-4CH_2_O-C_2_H_2_O-H_2_O-C_4_H_6_]^+^ | naringin dihydrochalcone |
| 27 | 10.40 | [M+H]^+^ | 223.06071 | 223.06015 | 4.5 | C_11_H_10_O_5_ | 223[M+H]^+^, 207[M+H-CH_3_]^+^, 191[M+H-2CH_3_]^+^, 164[M+H-2CH_3_-CO]^+^, 133[M+H-2CH_3_-2CO]^+^ | isofraxidin |
| 28 | 12.70 | [M+H]^+^ | 375.14402 | 375.14383 | 0.51 | C_20_H_20_O_7_ | 375[M+H]^+^, 275[M+H-C_5_H_8_O_2_]^+^, 260[M+H-C_5_H_8_O-CH_3_]^+^, 247[M+H-C_5_H_8_O_2_-CO]^+^, 233[M+H-C_5_H_8_O-CH_3_-CO]^+^, 221[M+H-C_5_H_8_O-C_4_H_6_]^+^, 205[M+H-C_5_H_8_O-CH_3_-2CO]^+^, 187[M+H-C_5_H_8_O_2_-CH_3_-2CO-H_2_O]^+^ | ledebomiellol |
| 29 | 12.90 | [M+H]^+^ | 233.04440 | 233.04445 | -0.2 | C_12_H_8_O_5_ | 233[M+H]^+^, 218[M+H-CH_3_]^+^, 190[M+H-CH_3_-CO]^+^, 173[M+H-CH_3_-CO_2_]^+^, 162 [M+H-CH_3_-2CO]^+^, 134[M+H-CH_3_-3CO]^+^, 116 [M+H-CH_3_-3CO-H_2_O]^+^ | 5-hydroxy-8-methoxypsoralen |
| 30 | 13.00 | [M+H]^+^ | 271.09660 | 271.09649 | 0.4 | C_16_H_14_O_4_ | 271[M+H]^+^, 215[M+H-2CO]^+^, 203[M+H-C_5_H_8_]^+^, 185[M+H-C_5_H_8_-H_2_O]^+^, 175[M+H-C_5_H_8_CO]^+^, 147[M+H-C_5_H_8_-2CO]^+^, 157[M+H-C_5_H_8_-CO-H_2_O]^+^, 137[M+H-C_5_H_8_-CO-CO_2_]^+^ | Imperatorin |
| 31 | 13.14 | [M+H]^+^ | 319.11753 | 319.11762 | 4.3 | C_17_H_18_O_6_ | 319[M+H]^+^, 259[M+H-C_2_H_4_O_2_]^+^, 244[M+H-C_2_H_4_O_2_-CH_3_]^+^, 229[M+H-C_2_H_4_O_2_-2CH_3_]^+^, 217[M+H-C_2_H_4_O_2_-C_2_H_2_O]^+^, 189[M+H-C_2_H_4_O_2_-C_2_H_2_O-CO]^+^, 205[M+H-C_6_H_10_O_2_]^+^ | 3-O-acetylhamaudol |
| 32 | 13.36 | [M+H]^+^ | 301.10740 | 301.10705 | -2.2 | C_17_H_16_O_5_ | 301[M+H]^+^, 245[M+H-C_4_H_8_]^+^, 233[M+H-C_5_H_8_]^+^, 230[M+H-C_4_H_8_-CH_3_]^+^, 218[M+H-C_5_H_8_-CH_3_]^+^, 217[M+H-C_5_H_8_-CH_4_]^+^, 215[M+H-C_5_H_8_-H_2_O]^+^, 202[[M+H-C_4_H_8_-CH_3_-CO]^+^, 190[M+H-C_5_H_8_-CH_4_-CO]^+^, 173[M+H-C_5_H_8_-H_2_O-C_2_H_2_O]^+^, 161[M+H-C_5_H_8_-CH_4_-2CO]^+^, 134[M+H-C_5_H_8_-CH_4_-3CO]^+^ | phellopterin |
| 33 | 14.40 | [M+H]^+^ | 347.14920 | 347.14892 | 0.8 | C_19_H_22_O_6_ | 347[M+H]^+^, 329[M+H-H_2_O]^+^, 259[M+H-C_4_H_8_O_2_]^+^, 244[M+H-C_4_H_8_O_2_-CH_3_]^+^, 231[M+H-C_4_H_8_O_2_-CO]^+^, 217[M+H-C_7_H_14_O_2_]^+^, 205[M+H-C_4_H_8_O_2_-CO-H_2_O]^+^, 189[M+H- C_7_H_14_O_2_-CO]^+^, 177[M+H-C_4_H_8_O_2_-2CO-H_2_O]^+^ | 3’-o-i-butyrylhamaudol |
| 34 | 17.59 | [M+H]^+^ | 227.17817 | 227.17819 | -0.5 | C_9_H_14_N | 227[M+H]^+^, 210[M+H-OH]^+^, 182[M+H-OH-CO]^+^, 211[M+H-NH_2_]^+^ | glycerate-3P |
| 35 | 17.59 | [M+H]^+^ | 268.20670 | 268.20671 | -0.5 | C_9_H_18_O_6_PN | 268[M+H]^+^, 251[M+H-OH]^+^, 234[M+H-2OH]^+^ | phosphoenolpyruvic acid |
| 36 | 20.59 | [M+H]^+^ | 355.07031 | 355.07059 | 2.53 | C_16_H_18_O_9_ | 355[M+H]^+^, 193[M+H-GLU]^+^,175[M+H-GLU-H_2_O]^+^, 150[M+H-GLU-H_2_O-CH_3_]^+^, 133[M+H-GLU-2H_2_O-CH_3_]^+^ | scopolin |
